# Effect of Coronavirus-19 on Mental Condition of International Students in China

**DOI:** 10.3389/fpsyt.2021.738828

**Published:** 2022-01-03

**Authors:** Faiza Manzoor, Longbao Wei, Muhammad Zia ul Haq

**Affiliations:** ^1^Department of Agricultural Economics and Management, School of Public Affairs, Zhejiang University, Hangzhou, China; ^2^Department of Business Administration, Air University Multan Campus, Multan, Pakistan

**Keywords:** COVID-19, emotional intelligence, psychological well-being, generalized anxiety disorder, China, international students

## Abstract

Despite its importance, no study investigates the effects of COVID-19 on the mental condition of international students in China. By using data from international students in China, this study finds the positive and affirmative connection between fear of COVID-19 and generalized anxiety disorder among foreign students. Furthermore, this study reveals a negative association between fear of COVID-19 and the psychological well-being of foreign students in China. We also find that emotional intelligence has a positive and optimistic moderating influence on the relationship between fear of COVID-19 and generalized anxiety disorder but it has not moderating effect on the relationship between fear of COVID-19 and psychological well-being. Our study contributes to the body and knowledge and offers new insights concerning the effects of COVID-19 on the mental condition of international students.

## Introduction

The COVID-19 pandemic has caused massive disruptions to everyone's life across the world. According to the World Health Organization (WHO), as of November 26th, there are 60, 074, 174 confirmed cases of COVID-19 with 1,416, 292 deaths worldwide (https://www.who.int/emergencies/diseases/novel-coronavirus-2019). The second wave of COVID-19 has started at the time of this study and the number of confirmed cases is increasing rapidly in all counties. The rise in the number of confirmed COVID-19 cases is also affecting students physically, academically, financially and psychological health (1). Many students in various universities have been tested Covid-19 positive (1). To stop the possible transmission of the virus, most of the universities have switched from physical to online teaching mode (2). The students are advised to vacate the campus and adapt to the online learning platform. Consequently, it has changed the lives of students dramatically (2). The shift to online mode has aggravated the level of stress in students given the reason these courses were not originally designed for online delivery (3). Moreover, students have concerns about their health and the health of their family members. Due to the prolonged closure of the service and retail industry, many students are also worried about their financial issues because they used to work part-time to support their studies. A recent survey has indicated that 4 out of 5 students are facing severe financial problems due to COVID-19 Pandemic (4). Additionally, students are not entitled to financial relief under the Corona Virus relief efforts.

Due to the fear of the COVID-19 pandemic, many international students are experiencing mental health issues such as psychological distress and anxiety disorder (5). The mental health issues may impair their social interactions and academic success influencing their future career goals (1). The measures taken to curtail COVID-19 such as social distancing and the rapid spread of the virus are further aggravating the mental health issues of people including international students. Previous studies have investigated the psychological impact of the COVID-19 on adults, workers, and the general public (6, 7). These studies have found an increased level of stress, anxiety disorder, and greater concerns about social isolation. According to Kecojevic et al. (1), accurate information acquisition about health during the COVID-19 pandemic are related to psychological well-being and lowers the level of stress among people (7). On the other hand, lack of information about health or inaccurate information is linked to severe mental stress and anxiety disorder (1). Scholars in the past have also suggested that following precautionary measures such as wearing the mask and frequently washing hands to contain the possible spread of COVID-19 may lower the anxiety disorder and improve psychological well-being by providing sense of security (7). Although universities are providing accurate information to international students, however, there is also a great risk of spreading inaccurate information through social media and online sources. Online sources and social media are often use by international students that may contribute to anxiety disorder among intentional students. Therefore, the situation calls for more empirical research to examine the linkages between fear of COVID-19 and psychological well-being, and anxiety disorder.

Previous studies have indicated that emotional intelligence (EI) is an important resource to improve the psychological well-being of students. It enhances the success of students learning and education quality (8). EI has a profound effect on mental health and reduces anxiety disorder. It increases the understanding and regulates the emotions among individuals. Previous studies have found that EI is also related to change in behaviors and improve the periodicity of people (8). Despite the significance of EI in improving psychological welling and reducing anxiety, there is a lack of explanation as to how EI moderates the relationships between Fear of COVID-19, Psychological well-being, and anxiety disorder.

The objective of this paper is to contribute to the body of knowledge by investigating the relationship between fear of COVID-19 and psychological well-being, and anxiety disorder among international students. It also contributes to the extant literature by explaining how EI moderates the relationships between fear of COVID-19, anxiety disorder, and psychological welling.

This paper structured as follows: First, the theoretical context and hypothesis related to fear of COVID-19, anxiety disorder, and psychological well-being are developed. Second, using data obtained from international students in China, the proposed research framework is tested. Finally, implications, conclusions, and recommendations for future research are given.

## Theoretical Background and Hypotheses

### The Impact of Fear of COVID-19 on Generalized Anxiety Disorder and Psychological Well-Being

Fear of COVID-19 has the potential to influence the mental health of international students (9). According to Barlow et al. (10), fear is a kind of emotion that is present across all ages including students. It is awareness and appraisal of anxiety and danger that occurs when fear is aggravated (10, 11). Fear of COVID-19 can form anxiety disorder very quickly among international students (12). Preventive measures that are taken to restrict the transmission of the COVID-19 virus has forced universities to switch to the online mode of teaching and learning. International students are advised to stay in their rooms and adjust and adapt to new living circumstances. Unfortunately, the courses were not designed for the online mode of teaching. Therefore, it enhanced the level of stress among international students. These courses require face-to-face interaction and lab work that was not possible in the online mode of teaching leading to a disadvantage to evaluate the performance of students. Students have also reported computer and internet problems while attending their classes online. According to Ahorsu et al. (12) with the availability of real-time information, wrong and inaccurate information on social media and online forums also create fear and anxiety disorders among people (12, 13). This fear has produced uncertainty about the future that leads to anxiety disorder among international students. Anxiety is a state of emotions that is generated due to threats and certainty about the future (12, 14). According to Epstein (15) anxiety poses threats to self-esteem, threats to happiness, and the future. We argue that uncertainty about the future may also impact students' quality of life resulting in anxiety disorder (16). This anxiety disorder may get momentum specifically among international upcoming graduates that are going to face the job market in near future. According to Asrar-ul-Haq et al. (17) University graduates between the age of 23 to 25 are in the process of developing their career commitments and expectations. However, their regular expectation level has come down as the global pandemic COVID-19 poses the highest threats to international students who are planning their careers for the future (18). Consequently, due to this uncertain situation international students' fear has turned toward anxiety disorder since they are unable to make suitable decision about their future careers. Therefore, we propose:

Psychological well-being refers to the state of satisfaction and happiness in one's life (19). It manifests the presence of positive emotions and the absence of negative emotions (19). People with psychological well-being possess good academic success, physical and mental health, skills social support, and an objective in life (19, 20). Previous studies have mainly focused on the well-being of the adult population in developed countries. Some scholars also paid attention to children's well-being in developing countries (21). These studies found that fear affects the psychological state of people (22, 23). We argue that fear of COVID-19 may affect the psychological well-being of international students during their studies while abroad. Fear can induce among international students many unwanted psychological states and increases insomnia, level of anxiety, depression, and mental health (24–26). The fear of COVID-19 may also badly affect other normal tasks of their life that, in turn, influence psychological well-being. Previous studies also endorse our argument that fear of COVID-19 strongly influences the psychological well-being of people (25, 27).

According to the conversion of resources theory, people secure and protect their important resources (28, 29). Resources can be intangible that carry their intrinsic value for the people such as health, self-esteem, attachments, and psychological well-being (29). The fear of COVID-19 transforms into worries of losing their valuable resources such as their health, the health of their family members and generates stress that consequently leads to depression, anxiety, and disorders (25, 29). Students with fear of COIVD-19 may also experience psychological and physical loss of resources that negatively impact their well-being (29, 30). Some scholars in the past have also drawn similar conclusions that fear losing resources leads to psychological strains among people. Therefore, we propose:

Hypothesis 1: Fear of COVID-19 is positively related to Generalized Anxiety Disorder.
Hypothesis 2: Fear of COVID-19 is negatively related to psychological well-being.

### The Impact of Emotional Intelligence on Generalized Anxiety Disorder and Psychological Well-Being

Emotional intelligence (EI) refers to the ability to perception and expression of emotions, assimilate emotions into meaningful thoughts, develop an understanding of reason with emotions and regulate the emotions in oneself and others (31–33). According to McShane et al. (33), EI has four dimensions. The first dimension deals with self-awareness. Self-awareness refers to an understanding of one's strengths and weaknesses, motives, and values. People with self-awareness are better able to respond to specific circumstances by managing and listening to their emotions (32, 33). The second dimension of EI is self-management. It refers to the ability to redirect and control the impulses and emotions and internal states (31–33). Similarly, social awareness and social management refer to the ability to understand the feelings, emotions, thoughts, and situations of others. It includes the management of other people's emotions (31, 32). People with relationship management skills can influence others, cultivate relationships, and resolve conflicts (33, 34).

EI has attracted the attention of scholars in recent times (33). The scholars have examined its role in business, education, and personal life (32, 35). While living abroad and studying, international students have to interact with their seniors, subordinates, teachers, and class fellows. According to Rathnakara (32), motivation and contentment are important factors to achieve higher performance in their studies. Student's attitudes will directly contribute to their academic success and psychological well-being including their mental health (36). World health organization (WHO) defines health as a state of complete mental, physical, and social well-being and in addition to the absence of disease. Well-being refers to the state of healthy, happy, and satisfied (36). According to Palmer et al. (36), well-being can be divided into three parts such as physical, psychological, and social. All these types of well-being are important for international students for their academic and future career success (32, 36). As indicated earlier, international students have to deal with different types of people while studying abroad. Therefore, their relationship with others can influence their state of well-being. EI is one of the most important factors in determining the quality of their interpersonal relationships. According to Mehta and Vasoo (37), the higher level of EI determines the quality of physical and psychological well-being. EI enables people to monitor the emotions and feelings of others, differentiate these feelings and emotions, and use these understanding to guide one's behavior and actions (37). Therefore, this type of EI allows to maintain a higher level of psychological well-being.

The relationship between EI and anxiety, depression, physical and mental health is well-documented in previous studies (37, 38). For example, people with greater attention to their emotions are better able to adjust their emotions as compared to people who have low emotional clarity (38). People with a deep understanding of their emotions have a higher possibility to regulate their state of emotions that leads to higher self-esteem which is an important indicator of mental health. Scholars in the past have provided evidence that EI intelligence leads to physical and psychological well-being (39). However, there is a lack of explanation of the relationship between EI and anxiety. Although, few studies focused on EI and social anxiety (39). Social anxiety occurs when a person is exposed to a stranger or scrutiny of others that create a constant state of fear for one or more performance or social circumstances. It creates deep anguish and restlessness and hinders psychosocial adjustment among individuals (40). Anxiety is people also lead to other mental health problems such as lineless and dysphoria and difficulty in maintain the interpersonal relationship with others (40). Moreover, international students were suffering from anxiety disorder may have avoidance behavior in response to their academic work that adversely affects their learning outcome. Consequently, they may present a lack of adaptability with their University and academic obligations. Additionally, people with a higher score in anxiety disorder often show victimization behavior in repose to bullying and cyberbullying (41, 42). Therefore, we propose:

Hypothesis 3: There is a negative relation between Emotional Intelligence and Generalized Anxiety Disorder.
Hypothesis 4: There is a positive relationship between Emotional Intelligence and Psychological well-being.

### Moderating Role of Emotional Intelligence Between Fear of COVID-19 and Generalized Anxiety Disorder and Psychological Well-Being

We argue that EI can play an important role in mitigating the negative effects of fear of COVID-19 on psychological well-being and anxiety disorder. According to McShane et al. (33), EI is the ability of a person to understand, regulate, express, and evaluate his own emotions and the emotions of others that guide the thinking and behavior to successfully face the challenges and demands of the environment. Previous studies have divided the construct of EI into four interrelated dimensions such as understanding of perception, facilitation, and regulation of emotions (43). It recognizes the ability and capacity differences among individuals and employs such understanding to regulate their actions and behaviors (43, 44). The extant literature suggests the regularization of emotions (44). This implies that a person can mitigate the negative effects of emotions by following certain emotional strategies when facing uncomfortable circumstances such as fear of CVOID-19 (45). Moreover, an individual can contribute and maintain his positive emotions in a negative situation by using specific strategies. Due to its importance, the idea of EI has attracted the attention of many scholars in the past (39, 46). According to Lau and Wu (47), EI is an important element of maturity that increases the psychological well-being of individuals. Moreover, EI is regarded as a fundamental factor in reducing anxiety disorder and improving positive well-being (48).

Moreover, resources conversion theory suggests that people try to preserve, protect, and pursue new resources when there is a threat of resource loss, an actual loss of resource, and a shortage of earned resource as a result of resource spending (49). According to resources conversion theory people use these resources to cope with stressful situations (28). Furthermore, additional resources can be developed and used more easily with the help of existing resources (49). We argue that EI as a resource enables students to manage generalized anxiety disorder and improves their psychological well-being. Because of their adaptive ability to assimilate emotional information and efficiently manage negative emotions and cognitions, students with a higher level of psychological resources such as EI are more tolerant of stressful conditions (49). EI allows them to endure less psychological stress and easily overcome the fear of COVID-19 by utilizing psychological resources reserves. Therefore, it is plausible to understand that EI may help students in finding the proper balance when using mental energy during studies in order to counter generalized anxiety disorder and improve psychological well-being. According to Lazarus (50) EI students are able to handle large amounts of emotional data quickly which in turn can help psychological adaptation. They consider environmental stressors and hurdles such as fear of COVID-19 as a challenge rather than a cause of stress, resulting in less aversive results for them (50). Previous studies also suggest that people with higher EI tend to focus on approaches that have worked in the past such as reliving happy memories and avoiding unproductive strategies such as avoiding difficulties. As a result, this can help to reduce generalized anxiety disorder and improve psychological well-being (49). EI can reduce the negative effects of COVID fear and enhances psychological well-being and reduces anxiety disorder by recognizing and managing their emotional reactions to the fear of COVID-19 (51). We further argue that a high level of EI in students will lead to a higher level of abilities to evaluate the emotional information and monitor their actions and the behavior of others that enables them to cope with the loss of resource, stress, and anxiety disorder due to the fear of COVID-19. Therefore, it is understandable that students evaluate the fear of COVID-19 differently according to their emotional capacity. More specifically, students with a higher level of EI are more likely to better evaluate, and regulate their emotions by identify the reasons for their feelings and will be less influenced by the fear of COVID-19 (52, 53). Previous studies also suggest that psychological and physical recovery after a disaster depends on the abilities of students to offset their loss (54). Similarly, Jordan et al. (52) found a negative relationship between EI and anxiety disorder while a positive relationship between EI and psychological well-being. Therefore, we propose:

Hypothesis 5: Emotional Intelligence will moderate the association between Fear of COVID-19 and Generalized Anxiety Disorder.
Hypothesis 6: Emotional Intelligence will moderate the association between Fear of COVID-19 and Psychological well-being.

## Methods of the Study

### Data Collection Procedure and Participants

**Figure 1:**
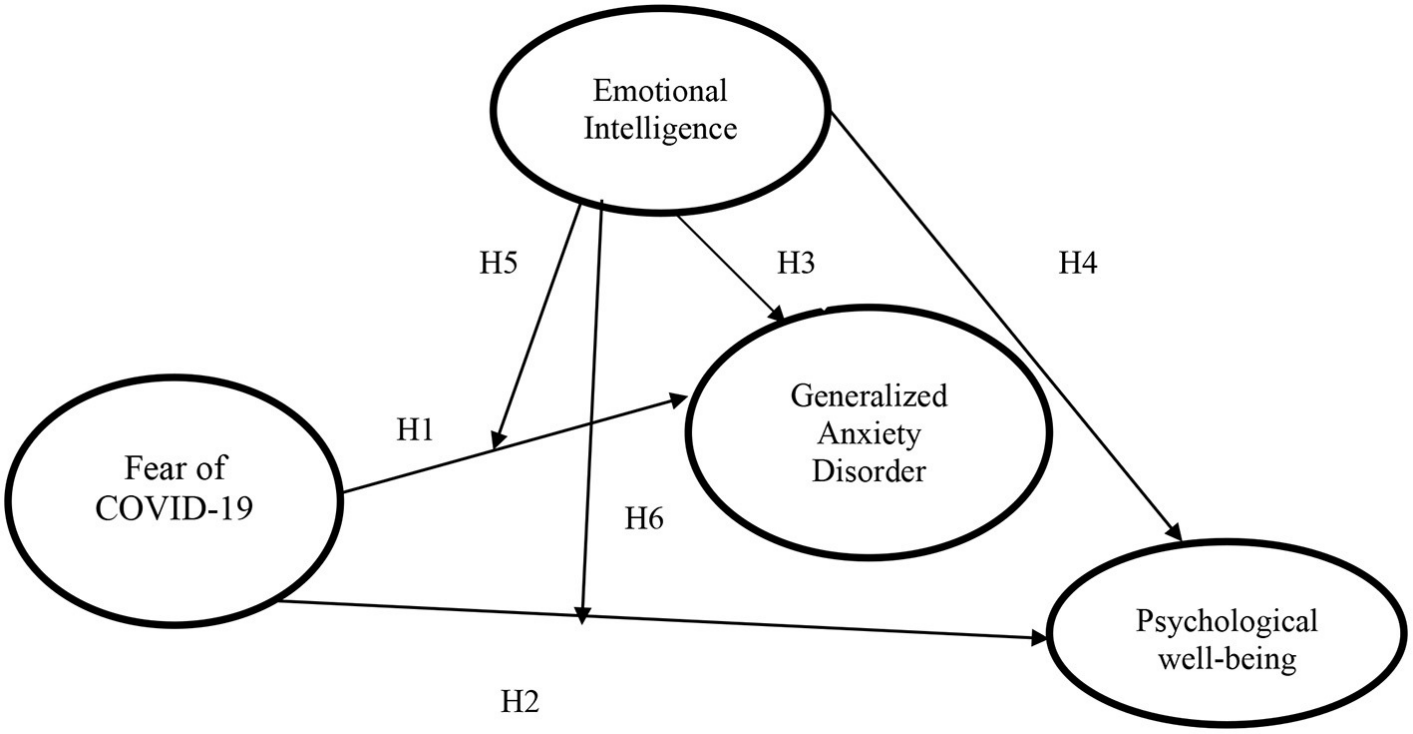
Conceptual study model.

The key objective of the present study is to determine the effect of fear of COVID-19 on the health condition of international students in China. Figure 1 showed hypotheses of the study and four main parameters. Fear of COVID-19 used as an explanatory variable, Generalized Anxiety Disorder and Psychological well-being are used as predicted variables as well as Emotional Intelligence is applied as a moderator variable. In general terms, a moderator is a qualitative or quantitative variable that affects the direction and/or strength of the relation between an independent or predictor variable and a dependent or criterion variable (55). Emotionally intelligent individuals can act appropriately with efficiently and effectively, and EI may provide them the capability to recover quickly from the aversive impact of fear (i.e., when it occurs), such that individuals of high emotional intelligence are less likely to experience fear. In the present study, we focus on the moderating effect of Emotional Intelligence to identify that EI may alleviate strain and depression resulting from fear.

The participants who contributed to this study were foreign students from different countries all over the world. They all came from different developing, developed nations, and currently studying in Chinese universities and academies. All they were in China during pandemics condition and faced lockdown and other difficulties (related to the epidemic). Without a doubt, the epidemic has affected everyone's lifestyles, work, and education, as well as how they interact with others. International students living in China were panic, had a difficult time as they were away from home country, and had a study stress and family pressure. They were unable to return to their home countries because international flights were prohibited during the pandemic time. They were suffering from acute mental depression due to a variety of factors. To get their mental condition and fear about COVID-19 we have conducted an online survey due to the lockdown situation. The Microsoft forms software platform was used for this online survey^1^ 380 respondents filled the online survey and we have evaluated their responses in our study. This survey consisted of all the study variables and included socio-demographic questions about age, gender, educational level, and nationality. The survey was conducted from early July to late September 2020.

Table 1 shows demographic characteristic of the survey respondents. This survey included 380 participants-52.6% (*n* = 200) male, and 47.4% (*n* = 180) female students from different nations (shown in Table 1). 25% (*n* = 95) of them were in the 25–30 age group, 59.5% (*n* = 226) were in between 31 and 39, and few participants 15.5% (*n* = 59) were 40 or above years old. Likewise, 42.1% (*n* = 160) were doctorate students, 41.1% (*n* = 156) were in master program, 8.4% (*n* = 32) were undergraduate students, and 8.4% (*n* = 32) participants belonged to others such as Chinese language students etc. Table 2 revealed the descriptive statistics, correlations and Cronbach alpha values of the study variables.

**Table 1:** Demographic valuation.

**Category**	**Frequency**	**Percent**
**Gender**		
Male	200	52.6
Female	180	47.4
**Age**		
25-above	95	25
30–39	226	59.5
40 or above	59	15.5
**Education**		
PhD	160	42.1
Master degree	156	41.1
Undergraduate	32	8.4
Other	32	8.4
**Nationality**		
Pakistan	35	9.2
Ukraine	19	5
Poland	9	2.4
India	8	2.1
Romania	8	2.1
Russia	9	2.4
Spain	8	2.1
Bangladesh	12	3.2
Malaysia	19	5
Korea	21	5.5
Indonesia	20	5.3
Sri Lanka	20	5.3
Nigeria	15	3.9
Sudan	24	6.3
Vietnam	35	9.2
Ghana	35	9.2
USA	50	13.2
Germany	33	8.7

**Table 2:** Reliabilities and Pearson's correlation.

***n* = 380**	**Mean**	**St. div**.	**Correlation**			
			**1**	**2**	**3**	**4**
1. FCV-19	3.899	1.147	**0.936**			
2. GAD	4.087	0.843	0.165**	**0.910**		
3. PWB	4.058	0.935	−0.203**	−0.032	**0.885**	
4. EI	3.514	1.33	0.171**	−0.255**	0.154**	**0.975**

*Correlation is significant at the 0.01 level (2-tailed). FCV-19, Fear of Covid-19; GAD, Generalized Anxiety Disorder; PWB, Psychological Well-being; EI, Emotional Intelligence. Bold values are reliability values*.

### Measurement of the Variables

This study adopted some measurement instruments; the key data collection tool was the seven-item fear of COVID-19 (FCV-19) (12, 56). The levels of agreement with COVID-19 statements were estimated at a five-point Likert scale from 1 (strongly disagree) to 5 (strongly agree). A sample item for FCV-19 is “I am very terrified of coronavirus-19.” The Cronbach alpha for the scale was 0.936.

The Generalized Anxiety Disorder (GAD) scale with seven-item was taken from the study of Spitzer et al. (57). The sample item is “Feeling nervous, anxious or even on the edge.” Each item is evaluated on a four-point severity scale (0 = not difficult at all; 3 = extremely difficult). GAD is a suitable instrument for screening for generalized anxiety disorder, evaluating the severity of anxiety symptoms over the past 14 days (last 2 weeks). Internal consistency alpha reliability of GAD was 0.910.

To measure psychological well-being (PWB) World Health Organization has developed a five-item scale, which is adapted from the study of De Wit et al. (58). It was conceptualized as a unidimensional measure that comprises five positively expressed items: e.g., “I felt in good spirits and cheerful.” The degree to which the aforementioned positive feelings were present in the last 14 days (2 weeks) is scored on a 6-point Likert scale ranging from 0 (not present) to 5 (constantly present). Cronbach alpha for PWB was 0.885.

Lastly, Emotional Intelligence (EI) was evaluated by using the sixteen-item scale initially developed by Wong and Law (44). An example item is “I always know whether or not I am happy.” The response format was a five-point Likert-type scale ranging from 1 = Strongly disagree to 5 = Strongly agree (21). Reliability estimates for the scale were 0.975. Cronbach's alpha reliability estimates for the scales are specified in Table 2 on the diagonals in bold. The alpha reliabilities were greater than the cutoff value of 0.70 (59, 60).

### Ethical Consideration

All procedures performed in the study involving human participants were in accordance with the ethical standards of the institutional research committee and with the 1964 Helsinki declaration. Consent was obtained from each participant.

## Results

Data analyses performed by the statistical package for Social Science (IBM-SPSS) v25.0, AMOS v.23.0 and Hayes process v.3 (61). The Hayes process is considered a more powerful and effective process than its alternatives (62), and 5,000 bootstrapping-based resamples have been selected.

Table 3 displays the outcomes of the confirmatory factor analysis of each study parameter. The findings showed that fear of COVID-19 has a good model fit as all model fit values are comparable to the maximum and minimum threshold values (63, 64). Emotional intelligence is a moderating variable in the present study; it displays a good model fit. Generalized Anxiety Disorder and psychological well-being are outcome variables. The findings of the one-factor analysis indicate that the predicted variables of the study have a good model fit. Furthermore, Table 3 reveals the overall model fit indices where the values of Chi-square goodness of fit test = 1150.83, Goodness of Fit Index (GFI) = 0.85, Adjusted Goodness of Fit Index (AGFI) = 0.83, Comparative Fit Index (CFI) = 0.94, Tucker Lewis index (TLI) = 0.95, Normed Fit Index (NFI) = 0.90, Root Mean Square Error of Approximation (RMSEA) = 0.05, and Standardized Root Mean Square Residual (SRMR) = 0.04. All values are excellent and met the threshold criteria (63, 65, 66). According to Bentler and Bonett (67), the estimates for CFI and NFI should be equal or higher than 0.9 for a good fit, while chi square /DF should be not more than 3. Manzoor et al. (68) and Asif et al. (69) recommended the estimates for NFI and CFI to be above 0.8 for a good fit.

**Table 3:** Confirmatory factor analysis.

**Latent variables**	**CMIN**	**DF**	**CMIN/DF**	**SRMR**	**GFI**	**AGFI**	**NFI**	**TLI**	**CFI**	**RMSEA**	**AVE**
Fear of Covid-19	36.034	9	4.004	0.024	0.969	0.927	0.979	0.97	0.984	0.089	0.677
Generalized anxiety disorder	56.271	14	4.019	0.031	0.957	0.913	0.966	0.96	0.974	0.089	0.604
Psychological well-being	8.699	5	1.74	0.012	0.991	0.973	0.991	0.99	0.996	0.044	0.608
Emotional intelligence	362.578	90	4.029	0.037	0.885	0.847	0.943	0.95	0.957	0.089	0.716
Overall model	1,150.83	554	2.077	0.048	0.854	0.834	0.906	0.95	0.949	0.053	

The values of Average Variance Extracted (AVE) lie between the recommended ranges (70, 71), specifying that the instruments used in the study have good validity.

Harman's one-factor test has been applied to test measurement biases (72, 73), which exposed that data does not suffer from the common method bias issue as the percentage of variance defined by a single factor is 39.86%, that is <50%. Hence, the study data is accepted as valid.

### Direct and Moderation Effect

Emotional intelligence has employed as a moderator variable in the study. A moderator variable is a third variable that impacts the intensity of the correlation between an outcome and an explanatory variable in a relationship, usually described as Manzoor et al. (74). Hayes process (v. 3) of the computer software IBM-SPSS (v. 25) was used to test the direct and moderation hypotheses for this study. The direct effect of fear of COVID-19 on Generalized Anxiety Disorder and Psychological well-being is stated in Table 4. The findings of analysis reveal that fear of COVID-19 has a significant and positive effect on Generalized Anxiety Disorder (estimated coefficient = 0.171, *P* < 0.01). Therefore, these findings are supporting hypothesis 1. Further, fear of COVID-19 and Psychological well-being has a negative and significant association (estimated coefficient = −0.198, *P* < 0.01). Hence, according to the results hypothesis, 2 is accepted. Likewise, the emotional intelligence with the value of (estimated coefficient = −0.170, *P* < 0.01) has a negative and significant effect on Generalized Anxiety Disorder. So, these findings support Hypothesis 3. Furthermore, emotional intelligence and Psychological well-being have a positive and significant association with (coefficient = 0.147, *P* < 0.01). However, hypothesis 4 is statistically accepted. As Table revealed that a lower level of confidence interval (LLCI) and upper level of confidence interval (ULCI) do not hold any zero, confirming that the direct relationship of the study variables is significant.

**Table 4:** Direct and moderating effect.

	**Coeff**	**SE**	**T**	**P**	**LLCI**	**ULCI**
**DV: generalized anxiety disorder**						
Constant	4.052	0.040	100.917	0.000	3.973	4.131
Fear of Covid-19	0.171	0.035	4.880	0.000	0.102	0.240
Emotional intelligence	−0.170	0.030	−5.601	0.000	−0.230	−0.110
Interaction I	0.131	0.025	5.156	0.001	0.081	0.182
**DV: psychological well-being**						
Constant	4.049	0.049	82.208	0.000	3.952	4.140
Fear of Covid-19	−0.198	0.043	−4.601	0.000	−0.283	−0.113
Emotional intelligence	0.147	0.037	3.958	0.001	0.074	0.220
Interaction II	0.035	0.031	1.137	0.255	−0.026	0.097

**Figure 2:**
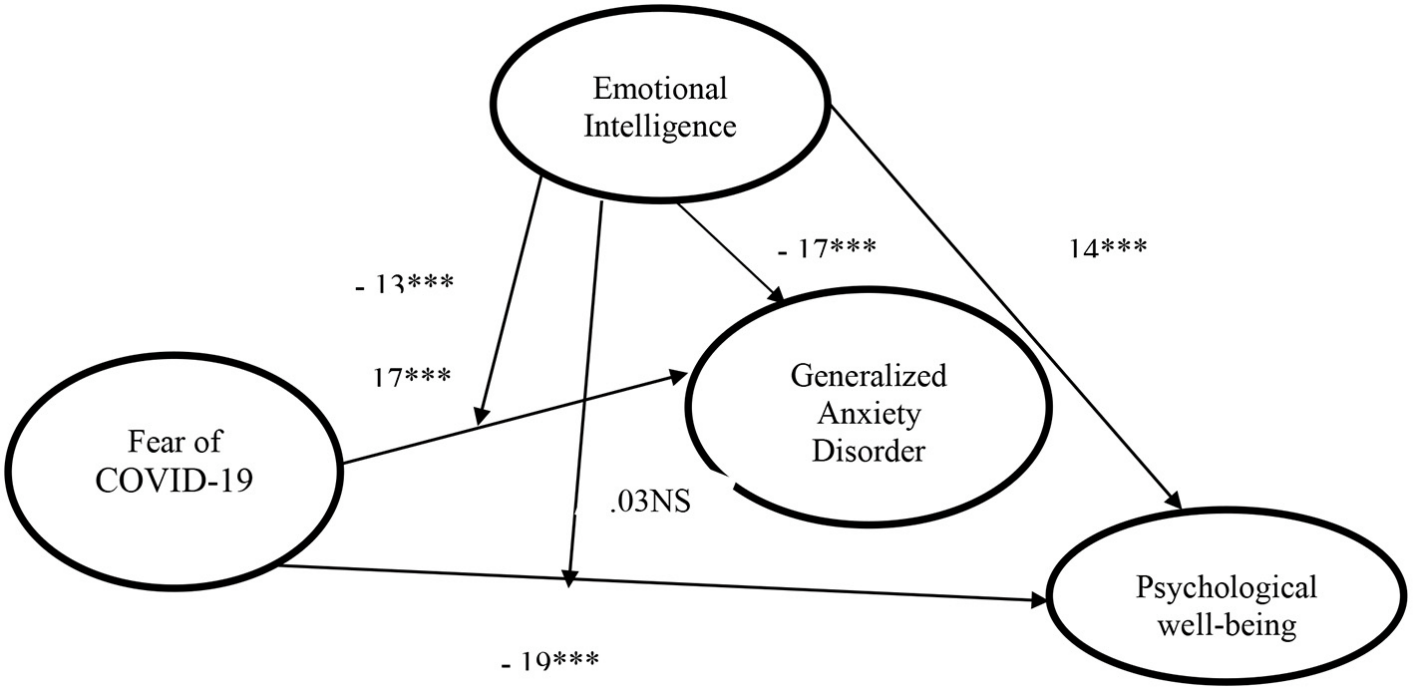
Results of the model, ***p < 0.001; NS, non-significant.

Hayes process moderation analysis is considered an important and more influential process than its alternatives to measure the moderation effect of the emotional intelligence on the explanatory variable and the outcome variables (62), as well 5,000 bootstrapping-based resample has been selected. Bootstrapping has no assumption of normal distribution. Also, Table 4 shows the outcome of the moderation effect (interaction I, interaction II). The result in Table 4 is supporting hypothesis 5 with the values of (interaction I coefficient = 0131, *P* < 001). It is stated that the relationship between fear of COVID-19 and Generalized Anxiety Disorder is moderated by emotional intelligence. On the other side, interaction II has an insignificant effect on the predicted variable (psychological well-being) with a *p*-value of 0.225 which is far higher than 0.05. Therefore, it can be stated that the relationship between fear of COVID-19 and Psychological well-being is not moderated by emotional intelligence. Moreover, Figure 2 also showed the direct and moderating effects of the variables.

**Figure 3:**
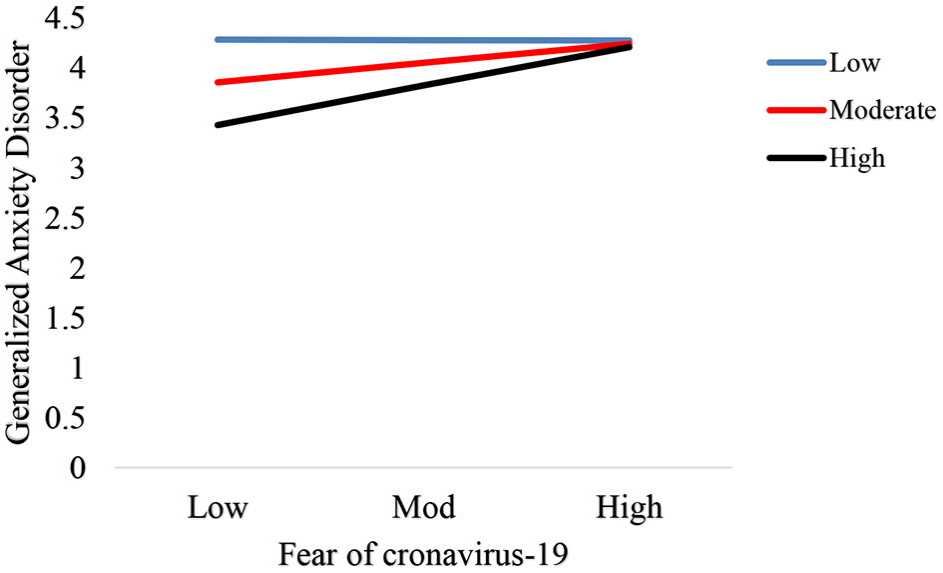
Interaction plot of the fear of COVID-19 and emotional intelligence on Generalized Anxiety Disorder.

**Figure 4:**
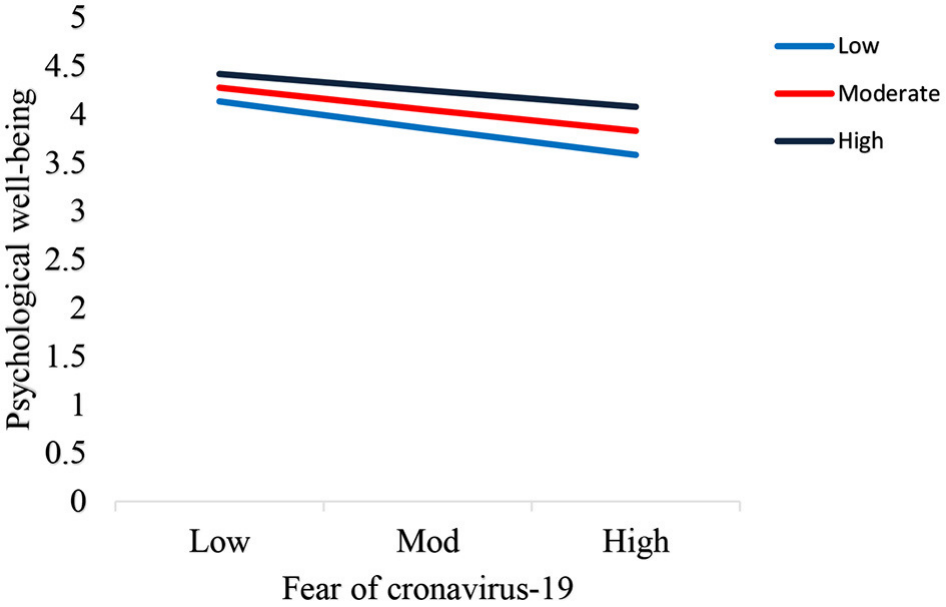
Interaction plot of the Fear of COVID-19 and emotional intelligence on psychological well-being.

The interaction graph for low, moderator and high values were plotted in this study. Figure 3 displays the interactive effect of the fear of COVID-19 and emotional intelligence (interaction I) on Generalized Anxiety Disorder. Figure 4 reveals the interactive effect of the fear of COVID-19 and emotional intelligence (interaction II) on Psychological well-being.

## Discussion

The prime purpose of this study is to empirically examine the fear of COVID-19 on the mental health of international students in China. In the study, we have used four main parameters such as fear of COVID-19 as an explanatory variable, Generalized Anxiety Disorder, and Psychological well-being as predicted variables. As well as this study observed emotional intelligence as a moderator variable between relationships of explanatory variable and predicted variables.

Previous studies showed that how fear of epidemics such as covid-19 increases depression, emotional distress, irritability, and insomnia (75, 76). The present fear level of overseas students is not different from Russian and Belarusian University students that reported by Gritsenko et al. (77), and Kazakhstan University students (56). This study explored the positive and affirmative connection between fear of COVID-19 and Generalized Anxiety Disorder of foreign students. In the pandemic situation, everybody was scared, and students who were far from their home countries and families were suffering a lot. In that situation they were terrified and confused; on the one side their family pressurized them to come back from China (because of outbreak country), and widespread media news, lockdown, number of increasing patients, and daily death rate were the cause of their anxiety disorder. On the other side, they were afraid of the loss of the academic year and study. Few of them were very close to their graduation, these kinds of situations made them very confused, and their anxiety level was very high. These findings are supported by the previous study by Konstantinov et al. (56) and Hetkamp et al. (78). Furthermore, this study reveals a negative association between COVID-19 fear and the Psychological well-being of foreign students in China. COVID-19 fear was diminished by the positive psychological well-being of the students. Positive psychological well-being tends to them lower fear of COVID-19 and higher satisfaction in life and study. Therefore, the results of COVID-19 fear and Psychological well-being are in the line with the previous study of Asad Ali Shah et al. (21) and Lopez et al. (79). Furthermore, this study showing that the moderator variable of the study emotional intelligence is also significantly and positively linked with the one outcome variable i.e., psychological well-being, and have a negative correlation with other predicted variable that is Generalized Anxiety Disorder. These outcomes are consistent with the results of the earlier research of Balluerka et al. (80), Guerra-Bustamante et al. (81), Lizeretti et al. (82), and Onur et al. (83), respectively. In addition, the results of the study shown that the expected first four hypotheses are completely acceptable.

Our study examined the moderating role of emotional intelligence between fear of COVID-19 and Generalized Anxiety Disorder as well as Psychological well-being, which is nearly non-existent in the health literature. However, this research studied this gap and confirmed that emotional intelligence has a positive and optimistic moderating influence in the relationship between fear of COVID-19 and Generalized Anxiety Disorder. Additionally, emotional intelligence has not moderating influence in the relationship between fear of COVID-19 and Psychological well-being. Moreover, the outcomes of the moderation analysis shown that the supposed hypothesis 5 is completely acceptable and hypothesis 6 is not.

To the best of our knowledge, this study is the first to identify the impact of COVID-19 fear on the mental condition of foreign students in China. Despite the cross-sectional nature of the study and the small number of survey respondents, which restrict the generalizability of the results, these findings tend to confirm that found in other countries. Moreover, and essential, this study outcomes prove a possible trend between overseas University students toward resilience and coping with current pandemic conditions—an important step toward a return to pre-pandemic living conditions. More inquiry is required across China universities (national plus international students) and overtime to develop a more thorough understanding of the COVID-19 impact on young adults connected to the development and future of the country.

## Data Availability Statement

The data presented in the study are included in the article/supplementary materials, further inquiries can be directed to the corresponding author/s.

## Ethics Statement

The studies involving human participants were reviewed and approved by Ethics Committee of Zhejiang University China. The patients/participants provided their written informed consent to participate in this study.

## Author Contributions

FM initiated the basic idea, wrote the main part of the manuscript, and built the article structure and Methodology. MH contributed to the literature review. LW reviewed and improved the manuscript. All authors contributed to the article and approved the submitted version.

## Conflict of Interest

The authors declare that the research was conducted in the absence of any commercial or financial relationships that could be construed as a potential conflict of interest.

## Publisher's Note

All claims expressed in this article are solely those of the authors and do not necessarily represent those of their affiliated organizations, or those of the publisher, the editors and the reviewers. Any product that may be evaluated in this article, or claim that may be made by its manufacturer, is not guaranteed or endorsed by the publisher.

## Footnotes

^1^ https://forms.office.com/Pages/ResponsePage.aspx?id=DQSIkWdsW0yxEjajBLZtrQAAAAAAAAAAAAO__TU2k2hUQlE4VjhFN0M4RjZUOEZVUTk5R0lVV0RSRC4u
